# A Haptotaxis Assay for Neutrophils using Optical Patterning and a High-content Approach

**DOI:** 10.1038/s41598-017-02993-6

**Published:** 2017-06-06

**Authors:** Joannie Roy, Javier Mazzaferri, János G. Filep, Santiago Costantino

**Affiliations:** 1 Research Center, Maisonneuve-Rosemont Hospital, Montreal, Quebec Canada; 2 Biomedical Engineering Institute, University of Montreal, Montreal, Quebec Canada; 3 Department of Pathology and Cell Biology, University of Montreal, Montreal, Quebec Canada; 4 Department of Ophthalmology, University of Montreal, Montreal, Quebec Canada

## Abstract

Neutrophil recruitment guided by chemotactic cues is a central event in host defense against infection and tissue injury. While the mechanisms underlying neutrophil chemotaxis have been extensively studied, these are just recently being addressed by using high-content approaches or surface-bound chemotactic gradients (haptotaxis) *in vitro*. Here, we report a haptotaxis assay, based on the classic under-agarose assay, which combines an optical patterning technique to generate surface-bound formyl peptide gradients as well as an automated imaging and analysis of a large number of migration trajectories. We show that human neutrophils migrate on covalently-bound formyl-peptide gradients, which influence the speed and frequency of neutrophil penetration under the agarose. Analysis revealed that neutrophils migrating on surface-bound patterns accumulate in the region of the highest peptide concentration, thereby mimicking *in vivo* events. We propose the use of a chemotactic precision index, gyration tensors and neutrophil penetration rate for characterizing haptotaxis. This high-content assay provides a simple approach that can be applied for studying molecular mechanisms underlying haptotaxis on user-defined gradient shape.

## Introduction

Neutrophils are rapidly recruited into infected or injured tissues where they play a pivotal role in host defense against invading pathogens and in wound healing^1^. Neutrophil trafficking into inflamed tissues is governed by chemotactic molecules secreted by invading bacteria and tissue-resident sentinel cells^2^ and coordinated expression of adhesion molecules on neutrophils and endothelial cells^3^. Since neutrophils encounter many chemoattractants, they prioritize and integrate chemotactic cues assuring preferential migration toward the inflamed site^4, 5^. Neutrophils recognize chemokines, such as CXCL8 (IL-8), which are thought to be bound to the surface of endothelial cells, forming a stable gradient to guide neutrophils toward the site of inflammation^6–9^. CXCL8 binds to the high affinity receptors CXCR1 and CXCR2 and evokes neutrophil chemotaxis via activation of the phosphoinositide 3-kinase-phosphatase and tensin homologue (PTEN) pathway^10^. Neutrophils change shape, polarize, crawl along concentration gradients and migrate across the endothelium into the tissue^11^. Formyl peptides of bacterial or mitochondrial origin^12^ through the formyl peptide receptors 1 (FPR1) and FPR2/lipoxin A_4_ receptor (FPR2/ALX) activate the p38 mitogen-activated protein kinase pathway, which dominates over the phosphoinositide 3-kinase pathway^10^ and direct neutrophils to arrive at the inflamed site^3^. Based on these characteristics, CXCL8 is classified as an intermediate chemotactic cue, whereas formyl peptides, such as N-formyl-Met-Leu-Phe (fMLF) a natural peptide or its synthetic analogs such as formyl-Nle-Leu-Phe-Nle-Tyr-Lys (fNLFNTK) are end-target cues^4, 13, 14^. In addition to formyl peptides, FPR2/ALX also binds a number of protein and lipid ligands that evoke opposing biological responses^15^. Importantly, ligand-specific conformational changes in FPR2/ALX pushes resolution of inflammation^16, 17^. Thus, a better understanding of neutrophil response to substrate-bound end-target chemoattractants opens novel opportunities for therapeutic interventions.

Several assays, including the under-agarose assay^10, 18, 19^ and filter-based assays, such as the Boyden chamber^6, 20, 21^ and the Zigmond chamber^22^ have been developed to study leukocyte migration *in vitro*. These assays commonly utilize temporally unstable gradients of soluble chemoattactants and therefore have limitations in mimicking haptotaxis *in vivo* ^23, 24^. Chemotaxis is traditionally characterized by end-point indices, like assessment of population-wide cell front migration distances^19^ or the fraction of cells that have migrated a predefined distance^6, 20, 21^. Over the years, chemotactic indices have been refined as tracking of individual cells became technically more feasible. It is also in the recent years that microfluidics-based migration assays were developed, allowing good gradient control^25, 26^, but being experimentally complex. Assays utilizing surface-bound guiding cues have traditionally been aimed at probing axonal guidance^27, 28^, but recent attempts have been made to apply these assays to investigate leukocyte migration^29–34^. For instance, Rink *et al*.^32^ used hydrogel-based microcontact printing to transfer CXCL8 gradients onto a petri dish surface to study neutrophil haptotaxis. While agarose stamping of a diffusion gradient is simple to perform, it results in only a short-lived protein gradient induced by the deposition of thin agarose layers on a substrate. The same group also used microcontact printing to immobilise CXCL8 on thiol-coated glass slide^29^.

Here, we report a novel high-content haptotaxis assay for neutrophils that is based on a modified version of the under-agarose assay, combining substrate-bound guiding cues, time-lapse imaging and a cell-tracking algorithm. We use laser-assisted protein adsorption by photobleaching (LAPAP)^35, 36^ to create reproducible substrate-bound formyl-peptide concentration gradients of varying steepness and shape. We can continuously monitor single cell migration patterns as well as perform statistical analysis of bulk migration along immobilized gradients. This assay reveals subtle haptotaxis differences that would have been difficult to observe without a high content approach. Combining the functional data with mathematical analysis evaluating chemotaxis indices currently in use, we propose penetration rate as a novel index for quantification of haptotaxis.

## Results

### High-content framework

**Figure 1:**
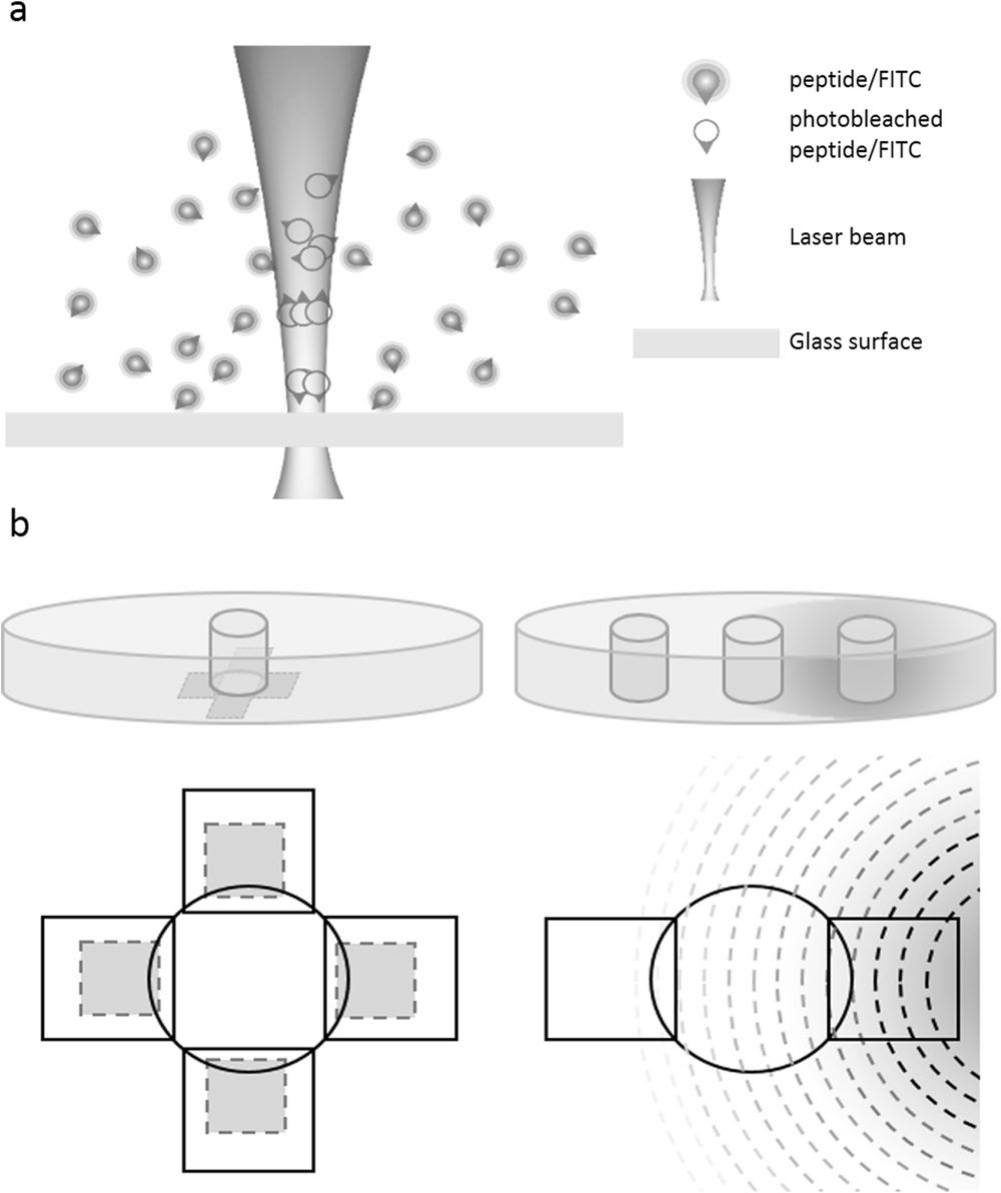
Schematic illustration of haptotaxis and classic under-agarose chemotaxis assay. (**a**) Optical patterning. The glass surface is incubated with FITC-tagged peptides. A focused laser beam photobleaches the molecules and binds it to the surface. (**b**) Side and top views of agarose-layered dishes (35 mm ø) with a central well (2 mm ø) to seed neutrophils. Top view of the central well shows the typical designs with multiple field-of-views alternatively imaged using a motorised stage. (*Left panel*) Substrate-bound gradients (shaded rectangles) are patterned at the bottom of the dish, on the glass surface, overlapping with the central well. (*Right panel*) A side well is filled with chemoattractant solution and the opposing side well with appropriate vehicle.

We have developed a framework for a high-content haptotaxis assay based on the well-established under-agarose assay (Fig. 1). In this approach, we control the camera, the light source and the motorised stage to acquire several movies in parallel and automatically track thousands of fluorescently labeled human primary neutrophils (Supplementary Videos S1 and S2). To avoid phototoxicity and photobleaching while accurately detecting and tracking cells, we carefully minimized the illumination and exposure during the assay. These adjustments were made by monitoring signs of toxicity such as morphological changes from amoeboid-like shape to round apoptotic cells (Supplementary Video S3).

### Optical patterning

**Figure 2:**
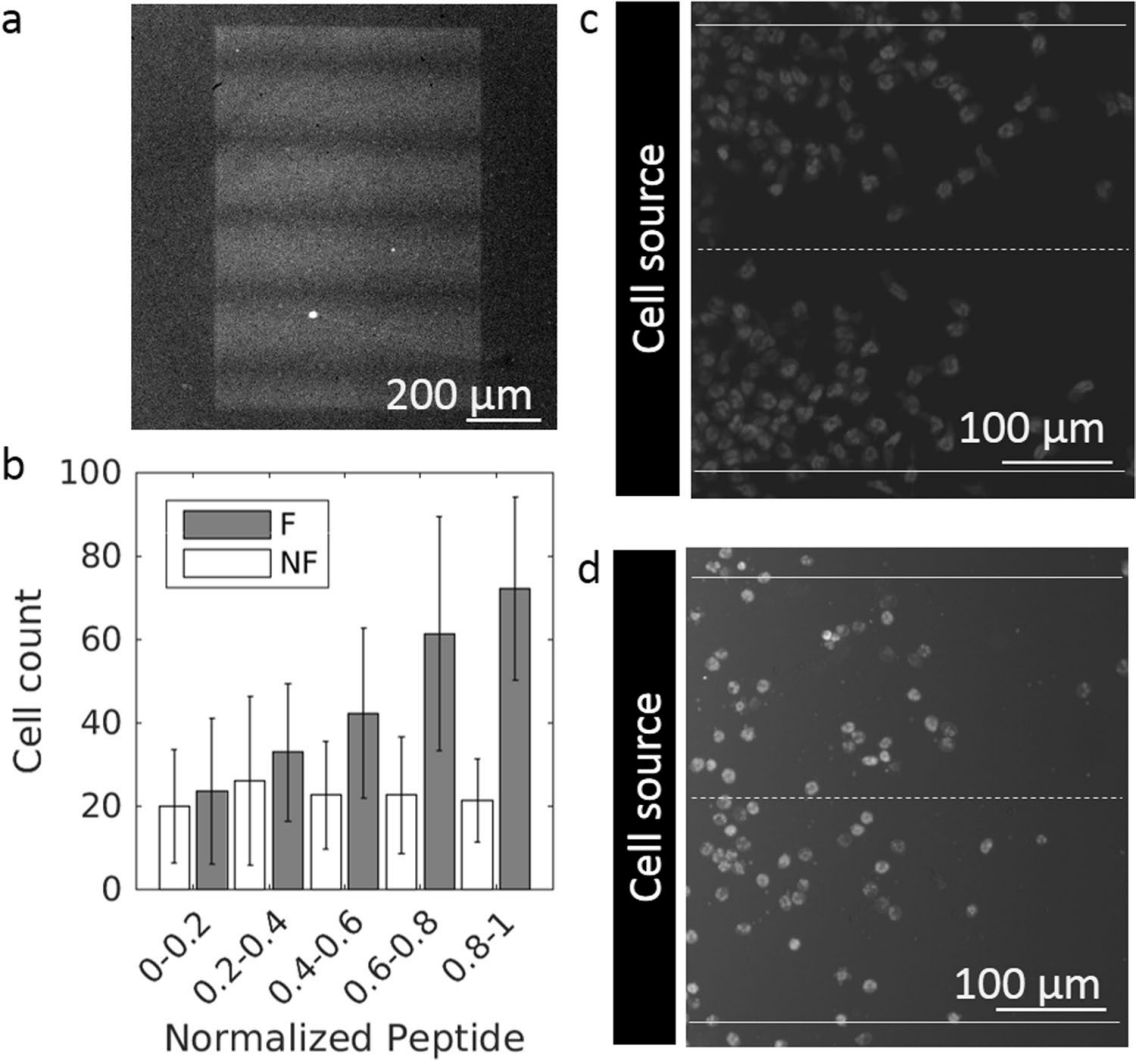
Neutrophil migration on substrate-bound peptide gradient. (**a**) Fluorescent image of typical surface-bound gradients. (**b**) Absolute cell count in binned regions of increasing normalized bound-peptide concentration of formylated (F) or non-formylated (NF) FITC-labeled NLFNTK. n = 3 replicates. Error bars represent SD. (**c** and **d**) Location of neutrophils on a formylated (**c**) or non-formylated (**d**) FITC-labeled NLFNTK pattern after 2 hours of migration. Dashed lines represent minima of gradient and plain lines maxima.

We modified the classic under-agarose assay, in which cells migrate toward soluble chemoattractant gradients, by micro-engineering substrate-bound gradients of a fluorescein-tagged formylated peptide (Fig. 1). LAPAP technology consists in varying the illumination power of a laser and moving the sample to tailor the geometry of the gradient produced by the photobleaching of the excited fluorophore^35^. We produced gradient of constant and continuous slopes to yield 4% and 8% difference in peptide concentration over the typical length of a cell (Supplementary Fig. S1). At saturation, this corresponds to an estimated 2.4 × 10^4^ molecules per cells (Supplementary Fig. S2 and Supplementary Methods). The pattern dimensions allow long and numerous cell tracks (Fig. 2a). After cells have migrated for two hours over the gradient, we detected a positive correlation between the cell density at any given position and the formyl peptide concentration (r = 0.97, p = 0.0078) (Fig. 2b and c, and Supplementary Video S2). There was no such correlation in the control assay of non-formylated peptide patterns (r = 0.53, p = 0.3554) (Fig. 2b and c).

### Detecting and Tracking neutrophils

**Figure 3:**
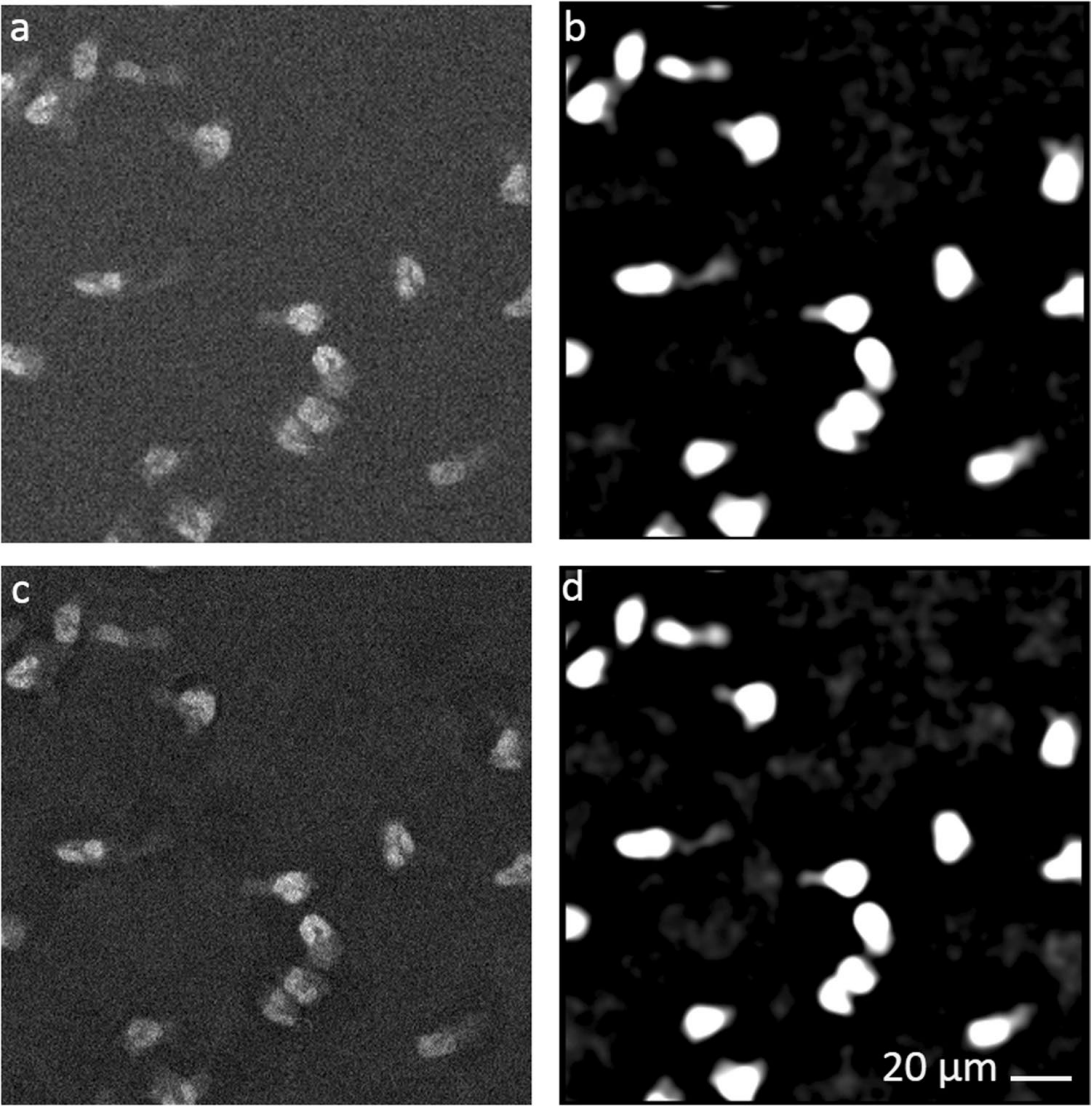
Cell detection by feature enhancement. Examples of the impact of different feature enhancement algorithms. (**a**) Original image. (**b**) Frequency filtering. (**c**) Background subtraction. (**d**) Background subtraction followed by a frequency filtering. Scale bar: 20 µm.

We processed the acquired data by a multi-step image analysis approach. In the pre-processing phase, the exact geometry of the gradients was registered. The cell detection algorithm consisted of a feature enhancement step, increasing the signal, followed by an object detection step (Fig. 3). Feature enhancement was performed by first subtracting a background image produced by using a morphological opening operation and then applying a frequency filter. The object detection was done by localising the high intensity peaks. Our approach shows a sensitivity in detecting cells with ~80% accuracy.

Cell tracking was done using a method that dynamically adapts to local cell density^37^. On average, we detected 148,580 trajectory steps per experiment (Supplementary Table SI). Typical tracks extend on average for 109 µm before cell arrest or truncation by the tracking algorithm. This yields an average of 2133 tracks/assay with neutrophils migrating at a speed around 10–20 µm/min.

### Chemotaxis indices

**Figure 4:**
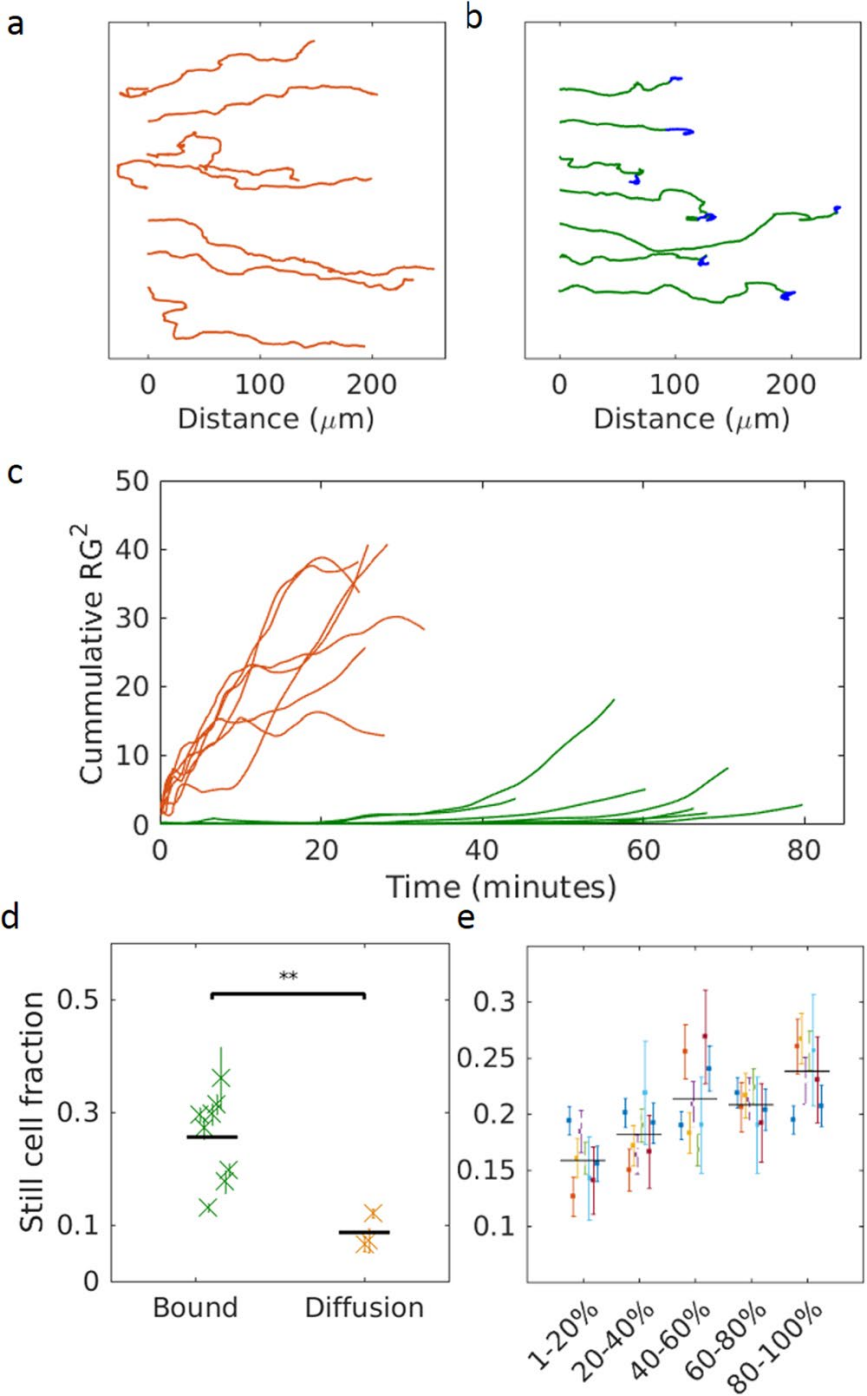
Comparison of migration track patterns. Representative tracks in the classic under-agarose chemotaxis assay (**a**) and substrate-bound haptotaxis assay (**b**). (**b**) The tracks are first persistent and elongated (in green), followed by deceleration and random walk around the end position (in blue). (**c**) Time-dependent evolution of the cumulative squared radius of gyration. Time 0 corresponds to the end of the track. Color coding is the same as in (**a**,**b**). (**d**) Fraction of total tracks showing dual behaviour (n = 3 for chemotaxis, n = 8 for haptotaxis assay). *p = 0.0065 (Student’s unpaired t-test). (**e**) Fraction of still cells in binned regions of gradients (n = 8 experiments). Colors represent individual experiments.

Neutrophils migrating under-agarose behave differently in response to soluble or substrate-bound formyl peptides. Under both conditions, neutrophils tracks are first elongated; cells moving away from their original position (the cell seeding well). However, a high fraction of cells moving on surface-bound formyl peptide eventually slow down and accumulate in the regions of high peptide concentration, still exhibiting amoeboid shapes but moving randomly around a central position (Fig. 4a and b, Supplementary Video S4). To describe this behaviour, we opted for an approach based on the gyration tensors of single cell track. Refer to Methods for a detailed explanation of the tensors. Intuitively, the gyration tensor provides a geometric representation of the distribution and shape of the coordinates defining a trajectory. The gyration radius (Rg) represents the spread of the cell locations, while the parameter A_2_ indicates the degree to which the trajectory shape is linear. By studying the temporal evolution Rg (R_g_^2^_N_(t)) for each track, starting from the end of the track, we detected this dual behaviour (elongation followed by random walk) of cells migrating on substrate-bound gradient (Fig. 4c). Indeed, while cell positions spread monotonically in the classic under-agarose assay, cell positions plateau on substrate-bound gradients. As the graph shows the tracks from end to the start, the plateau (in green) is a very gentle slope lasting for at least the last half of the tracks. In the diffusion assay, the evolution of the slope is more linear and steeper. Thus, in haptotaxis assays, 31% of neutrophils eventually move randomly around an end position within 2 hours of migration as opposed to only 10% of the cells migrating along a soluble formyl peptide gradient (Fig. 4d). Truncating the standstill part of these tracks, we detected a positive correlation between the concentration of the peptide and the extracted centroid (Fig. 4e). Combining the results of 8 assays, we computed a Pearson correlation coefficient of 0.77 (p = 4.39 × 10^−9^).

**Figure 5:**
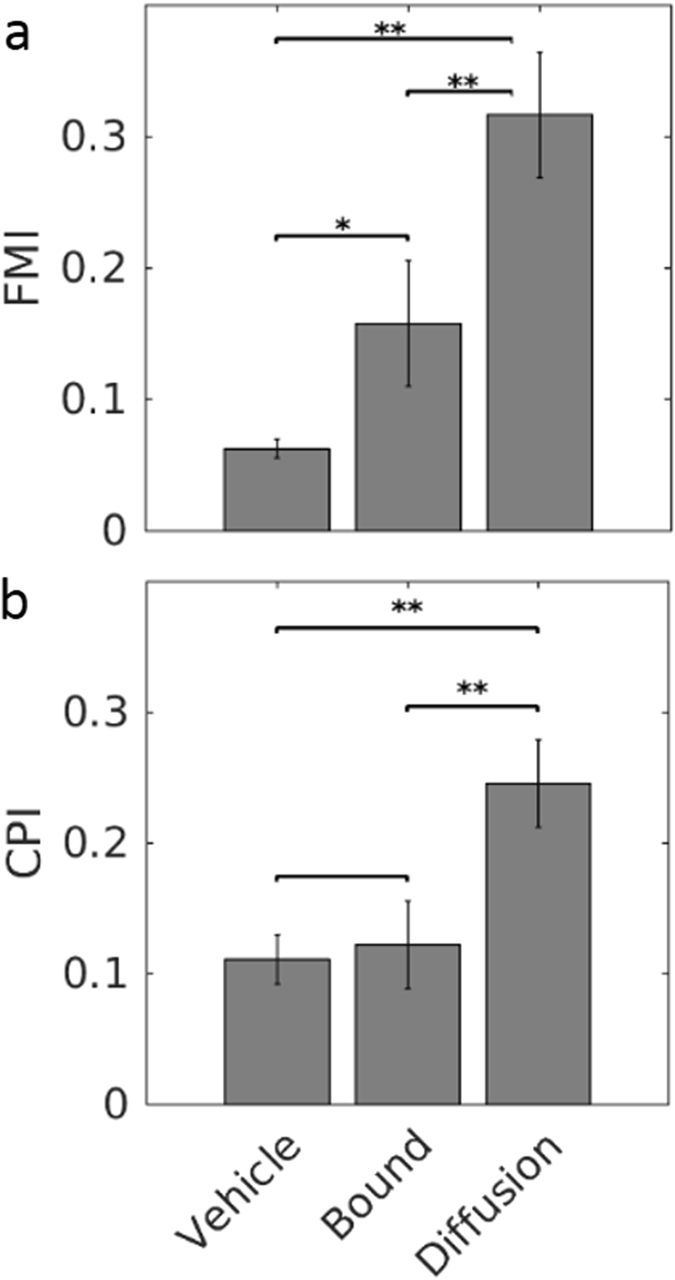
Comparison of indices of migration. (**a**) Forward Migration Index and (**b**) Chemotactic Precision Index have been calculated for neutrophils migrating toward a soluble gradient of non-chemotactic solution (Vehicle), soluble fNLFNTK (Diffusion) or on a substrate-bound gradient of fNLFNTK (Bound). *p < 0.01; **p < 0.001 (Unpaired Student’s t-test). n = 2 for Vehicle, 2 for Diffusion and 8 for Bound.

Rink *et al*.^32^ recently suggested characterising chemotaxis by calculating the Chemotactic Precision Index (CPI) instead of the more common Forward Migration Index (FMI, also known simply as the Chemotactic Index or as the McCutcheon Index)^38–40^. They claim that CPI is a better indicator of chemotaxis because it reports simultaneously on three aspects of the cell movement: it increases as the cell moves directly toward an end point (directness), it increases if the movement is in the gradient direction (cos^*2*^ *ϕ*, where ϕ is the angle between FMI and directness), and it decreases if the displacement diverts in other direction (1-|SMI|, where SMI stands for Side Migration Index). We tested the robustness of both FMI and CPI on data from of our haptotaxis assay as well as the classic under-agarose assay. In the latter assay, both indexes increased when neutrophils migrated towards a soluble fNLFNTK/FITC gradient versus the non-chemotactic control (Fig. 5). When cells migrated on surface-bound formyl peptides, since we oriented the gradient perpendicular to the radial axis of the seeding well, we expected that the intrinsic movement of cells moving radially away from the well should balance the migration in the gradient direction. Indeed, while the FMI, which considers only the forward force, was falsely elevated, the CPI was low, since both side and forward forces balanced each other.

### Speed and Orientation Dynamics

**Figure 6:**
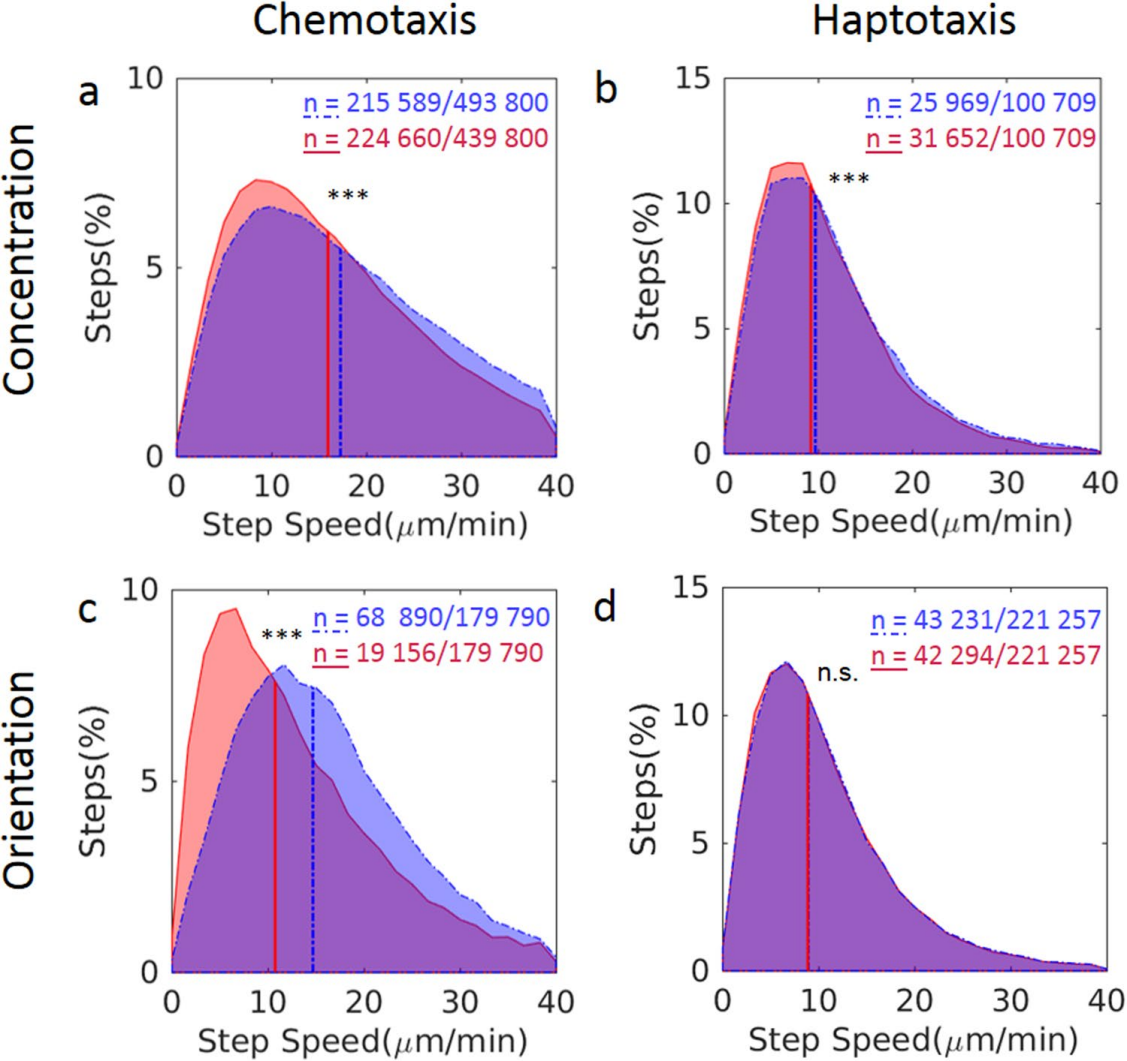
Trajectory step speed distribution of migrating neutrophils. Neutrophil migration in diffusible gradient (**a**,**c**) and on substrate-bound formyl peptide gradient (**b**,**d**). (**a**,**b**) Speed distribution of neutrophils migrating in low (red) or high (blue) peptide concentration part of the gradient. (**c**,**d**) Speed distribution of trajectory steps oriented relative to the gradient (blue) and opposed to the gradient (red). *** p < 0.001 (Wilcoxon-Mann-Whitney test). n.s., not significant. n = number of steps in a category out of total number of steps.

To investigate whether the correlation between cell density distribution and the gradient concentration was due to changes in cell speed (orthokinesis) or in turning angle (klinokinesis), we compared the distribution of speed and orientation of all trajectory steps. Regardless of their relative orientation to the gradients, neutrophils always moved faster in regions of high peptide concentration. In the classic under-agarose assays, the velocity was approximately 20% (n = 2) higher in the high fNLFNTK/FITC concentration regions than in the low ones (Fig. 6a). Although the correlation was subtler in the haptotaxis assays, neutrophils also migrated significantly faster on the high peptide concentration regions (mean value of 5.7%, n = 8) (Fig. 6b). This correlation was slope dependent, since the differences in speed were more pronounced for steeper slopes than gentler ones (Supplementary Fig. S3). Likewise, in the classic chemotaxis assays, we detected a strong correlation between the speed and the orientation of the cell to the gradient (Fig. 6c). For the sake of simplicity, we considered the soluble gradient to be linear and we defined a trajectory step as oriented if it was within 45° of the gradient direction. A step was considered as opposed to the gradient if it was within the opposite 45° of the gradient direction. In the haptotaxis assays, we couldn’t detect a correlation between the step speed and orientation (orthotaxis) (Fig. 6d).

**Figure 7:**
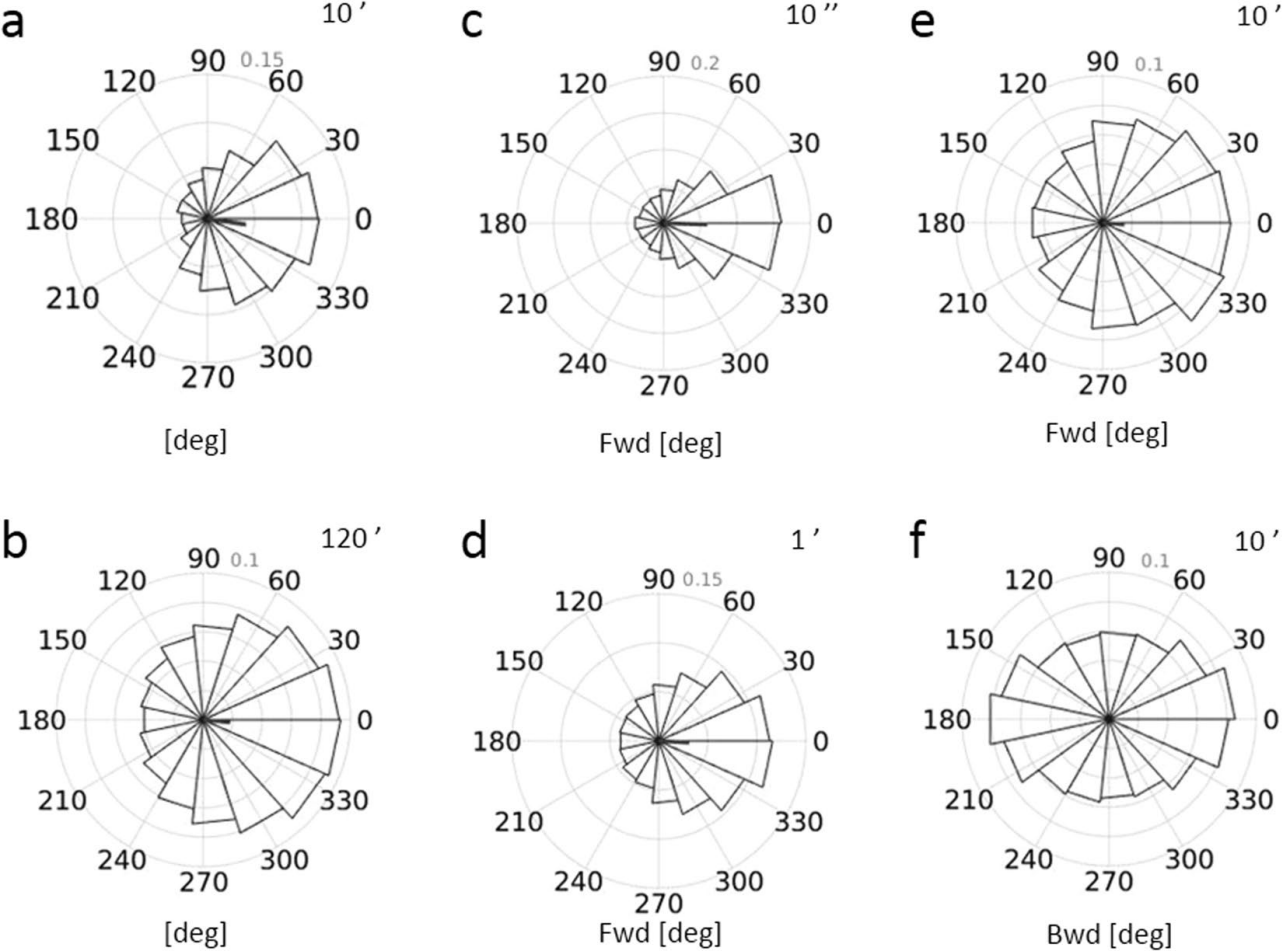
Cell orientation dynamics in the absence of chemoattractant. (**a**,**b**) Distribution of step angles over 10 minutes (**a**) or 120 minutes (**b**). The distributions are skewed to 0° (direction away from the well), especially at the beginning of the assay. (**c**–**f**) Distribution of step angles differences after an initial forward step (**c**–**e**), or backward step (**f**) for delay times of 10 seconds, 1 minute and 10 minutes, respectively. The results are representative for 8 independent experiments.

In the absence of chemoattractant, analysing the distribution of cell orientation in time, we observed that neutrophils were preferentially oriented radially to the seeding well. This observation holds true especially at early time points in the migration assay, as neutrophils spread out more randomly with time (Fig. 7a and b). We also measured the persistence of cell orientation in time by analysing pairs of step angle separated by different time lags. Figure 7c,d,e and f depicts the distribution of step angles classified as cells going forward or backward for 10 seconds, 1 minute or 10 minutes delays, respectively. A cell was defined as moving forward when its step was within [−45°, 45°] of the orthogonal direction of the well edge, pointing away from it. If a cell was initially going forward, it had a higher probability to be pointing in the same direction at a later time point, as indicated by the skewed distribution in 7c, d and e. On the other hand, if a cell pointed backward [135°, 225°], it had equal chances to be pointing backward or forward later on (Fig. 7f).

### Penetration rate

**Figure 8:**
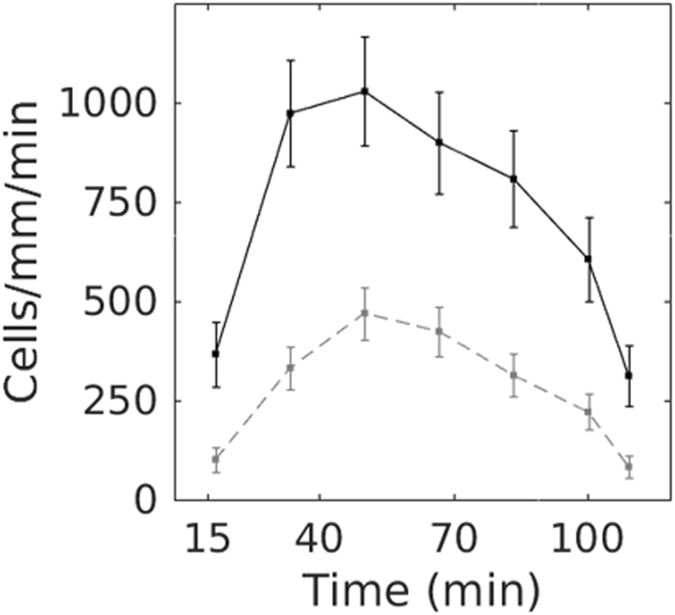
Neutrophil penetration rate into the under-agarose space. The number of cells exiting the well was determined at different time points and normalized by wall width and time interval. Black and gray (dotted) curves depict cells located on a homogenous saturated formyl peptide pattern (n = 272) and on 20% of saturation pattern (n = 211), respectively. Neutrophils exited the well at a higher rate when located on a pattern with high formyl peptide concentration (P = 6.2 × 10^−16^, χ^2^ test). Values represent the mean ± SD. Results are representative for 3 independent experiments.

To further characterize the correlation between neutrophil location and substrate-bound peptide concentration, we calculated the rate at which cells left the well to penetrate the under-agarose space. Comparing the number of tracks initiated on two 400 μm wide adjacent homogenous patterns of high (at saturation) or low concentration (20% of saturation) over 2 hours, we found that neutrophils exited the seeding well at a considerably faster pace when they moved over the high concentration pattern (Fig. 8 and Supplementary Video S5).

## Discussion

We used an optical patterning technique to generate surface-bound formyl peptide gradients with precision of approximately 1 µm, determined by the diffraction limit^35^. Alignment of gradients to the edge of the cell seeding well and time-lapse imaging allowed the analysis of a large number of migrating neutrophils under diverse conditions. As opposed to the Boyden, Zigmond and Dunn chamber assays, optical patterning results in well-defined surface-bound gradients that are highly reproducible in shape. The stability of the gradient is ensured by the covalent peptidic cross-link to surface, thereby preventing the formation of a soluble gradient in close proximity to the surface, which might happen using the hydrogel stamping technique^32^. Although *in vivo* neutrophils are likely exposed to both immobilized and soluble gradients, temporal fluctuations can complicate interpretation of results from *in vitro* migration assays.

Our results show that, in addition to soluble or stamped formyl peptides, neutrophils sense and respond to covalently bound cues. The average velocities detected in the high concentration areas are larger than in the low concentration areas of the immobilized gradient. Such high velocities were observed until the cells change from haptotaxis to haptokinesis. At this stage, the trajectories of cells are random, of low velocity and centered on an end-point. The observed dual behaviour, mimics the *in vivo* situation where neutrophils stop as they reach their end target^41–43^. We propose using the squared radius of gyration to describe neutrophils accumulating at the end targets. This parameter is particularly suitable to assess the shape of a trajectory in time.

Our modification to the classic under-agarose assay allows a simple way to test arbitrary protein patterns. The slope ranges were chosen from previous studies that showed that neutrophils are sensitive to 1–10% differences in concentration^22, 44^. Neutrophils exhibited more robust responses to steep gradients than gentle ones. These findings are consistent with a previous study, which reported a positive correlation between responses to CXCL8 and the slope of the gradient^45^. Furthermore, neutrophils were reported to preferentially migrate on a steeper gradient in bifurcating microfluidic channels containing different chemokine gradients^46^.

Neutrophils chemotaxis is governed by multiple cues generated by infectious agents and resident immune cells, including the neutrophils themselves. In our assay, we found that neutrophils tend to move away from the well, resulting in a bias in the forward direction even in the absence of a chemoattractant or when the gradient was perpendicular to the forward direction. This suggests that neutrophils might secrete chemokines in the seeding well, thereby confounding the influence of the immobilized gradients. While such chemorepulsive action has long been recognized, it was considered to have minimal impact at standard cell density in the under-agarose chemotaxis assay^19, 47^. However, our results (Fig. 7 and Supplementary Figures S4 and S5) indicate that at higher cell densities (such as that inside the well), neutrophil-secreted chemokines, such as CXCL8, might reach sufficiently high concentrations to evoke chemorepulsion via paracrine signalling^48^. Indeed, CXCL8 at low concentrations is chemoattractant for neutrophils, where as it becomes repulsive at concentrations higher than 1 µM^49^. This biased movement seems to differ from reverse migration, which has been attributed to desensitization to the chemoattractant^50^. Strong chemorepulsion may also explain why we failed to observe a clear preferential orientation of cell movement to the substrate-bound gradient oriented perpendicular to the well radius, regardless of the time of exit or the distance from the well (data not shown). A weak preferential orientation can be extrapolated from Supplementary Fig. S5e, where the cells are pulled sideward from their initial orientation at 10-minutes interval. However, in all other cases, they are oriented persistently in time. The chemorepulsion effect observed is also consistent with the lack of significant difference in CPI vs. vehicle control. The distinct responses to soluble and surface-bound formyl peptides can be explained by differences in the topographic distribution, association/dissociation, dimerization or internalisation rate of the formyl peptide receptors, though these events remain to be investigated^51, 52^. Hoffman *et al*.^51^ calculated expression of up to 71 000 receptors/cell, depending on receptor regulation, which is in the same order of magnitude of our calculated 2.4 × 10^4^ substrate-bound molecule/cell (assuming a cell size of 10 μm × 10 µm). For comparison, exposed to 50 nM fMLF in solution, for a volume of 10 × 10 × 10 μm, a cell would perceive 3.0 × 10^4^ diffusing molecules. While this manuscript was under revision, Schwarz *et al*.^33^ reported a similar LAPAP patterning gradient of CCL21 and the calculated number of molecules/μm^2^ was in the same order of magnitude as in our study.

We propose the penetration rate as an index describing the dynamics of the cells exiting the well and entering the under-agarose space. We explain the correlation between the neutrophil density and gradient concentration shown on Fig. 4 by the modulation of the penetration rate to the peptide concentration combined with cell accumulation at end points, as described by the gyration tensor.

In summary, we developed a novel high-content under-agarose assay for studying haptotaxis *in vitro*, where the geometry of the surface-bound chemical gradients can be controlled with micrometric precision. Our assay offers the advantage of studying 3D events in a 2D plane by confining neutrophils in space, thereby mimicking interstitial cell movement such as integrin-independent migration^34, 46, 53, 54^. These assays are of particular interest when studying low adhering leukocytes such as neutrophils or dendritic cells^34^. While in this study we fabricated formyl peptide gradients bound to BSA-coated glass surface, LAPAP can be performed to immobilize other small molecules, like chemokines, to many matrices^33, 34, 36^. To successfully immobilize other cues than fMLF, one has to carefully design the binding moiety, taking into consideration the inherent adhesiveness of the molecule to the substrate and the proper presentation of the binding domain to the cell surface receptors^33, 34^. Furthermore, LAPAP can produce continuous patterns, which could facilitate density-dependent cell spreading and/or adhesion studies. LAPAP represents an alternative to microcontact printing and recently has also been applied to microfluidic platforms^33^. Chemoattractants may have anionic or hydrophobic sites that are potential areas for surface binding^55^. Thus, extracellular matrix-bound CXCL8^55^, surface-bound PAF^56^, C5a^7^ and exosomes presenting LTB4 gradients^57^ were reported to evoke haptotaxis. fMLF can bind to albumin^58, 59^, to neutral endopeptidase (EC 3.4.24.11)^60^ and contribute to the association of neutrophils to fibrin and plasma clots^61, 62^, triggering migration. Since fibrin, albumin and neutral endopeptidase are present within inflamed or injured tissues^63^, it is plausible to assume that immobilization of fMLF does occur. Neutral endopeptidase cleaves formyl peptides and therefore is well-suited to modulate local levels. We are unaware of any direct demonstration of gradients of surface-bound chemoattractants extending from endothelial junctions through the intact to inflamed tissue *in vivo*. However, recruitment of neutrophils to inflamed tissues is likely regulated by subsequent chemoattractant gradients with formyl peptides being responsible for the final step of migration into the damaged area^3, 4^. We believe our assay presents a haptotaxis design, where patterning fMLF can be used for the assessment of neutrophil migration as a proxy for pathology^64, 65^. With our imaging and data processing system we can study a high number of cell trajectories, revealing previously unknown subtleties of haptotaxis. Thus, we showed that human neutrophils do respond to covalently-bound formyl peptide gradients. Neutrophils exit the well preferentially in the regions of high peptide concentration (penetration rate). In these regions, cells first speed up and suddenly stop migrating (although they continue to move randomly) and end up accumulating, passing from a haptotaxis behaviour to haptokinesis. This behaviour of neutrophils accumulating on surface-bound patterns mimics the *in vivo* situation where cells stop migrating after reaching their target. Our results also document a chemo-repulsive effect originating from the high cell concentration in the seeding well. Finally, as differences in neutrophil responses to soluble and surface-bound gradients are being increasingly recognized, our high-content assay provides a straightforward and inexpensive approach for studying haptotaxis and the underlying molecular mechanisms under conditions that mimic the *in vivo* situation.

## Methods

### Neutrophil isolation and staining

Neutrophils were isolated^66^ from the venous blood of healthy volunteers who had denied taking any medication for at least 2 weeks. In brief, the isolation method is based on the standard Bøyum procedure^67^ using lithium heparin as an anti-coagulant. On regular basis, we have monitor parameters indicating activation such as L-selectin shedding, CD11b upregulation, superoxide/ROS formation and found negligible or no activation during our isolation procedure. Neutrophils (5 × 10^6^ cells/ml, purity >96%, viability >98%) in Hank’s balanced salt solution supplemented with 10% autologous serum were stained with the dsDNA dye LDS751 (Molecular Probe) for 5 minutes at 37 °C^68^. The cells were then washed and resuspended in RPMI 1640 medium containing 5% FBS. 2.5 × 10^5^ cells were used per experiment. All methods were performed according to the Maisonneuve-Rosemont Hospital guidelines and regulation. The Clinical Research Committee of the Maisonneuve-Rosemont Hospital approved the experimental protocols. The authors confirm that written informed consent was obtained from all subjects.

### Optical patterning of substrate-bound gradients

We fabricated the haptotactic gradients optically, using a variation of LAPAP (Fig. 1a)^36, 69^. In brief, formyl-Nle-Leu-Phe-Nle-Tyr-Lys coupled with FITC (fNLFNTK/FITC, Molecular Probe) or a custom-synthesized negative control non-formylated peptide of the same sequence (H_2_N-Nle-Leu-Phe-Nle-Tyr-Lys/FITC, New England Peptide, USA) at a concentration of 0.5 mg/ml was incubated on BSA-coated glass bottom culture dishes (MatTek). The peptide was cross-linked by focusing a 473 nm laser (waist before the lens ~1 mm) on the glass surface with a 38 mm focal length lens. At this wavelength, the fluorophore photobleaches, liberating free-radicals that facilitate the adsorption of the peptide at the surface. We produced the concentration gradient by varying the power of the laser between 40 µw (minimal condition for peptide adsorption) and 400 µw (saturated peptide adsorption). We designed the shape of the pattern by moving the sample with a motorized stage at 0.1 mm/s controlled by a custom-made LabVIEW software (National Instruments, TX, USA).

### Under-agarose chemotaxis and haptotaxis assays

Low-melting agarose (UltraPure), solubilised in Earle’s Balanced Salt Solution (EBSS, Gibco) and brought to a 3% final concentration in RPMI 1640 medium supplemented with 10% FBS (Gibco) was poured onto glass-bottom culture dishes to obtain a ~3 mm layer. Upon gelling, a cylindrical piece of agarose of 2 mm in diameter was removed with a chirurgical punch to seed neutrophils, corresponding to a volume of 10 µl. For the haptotaxis assays, the peptide gradients were patterned on the glass surface before pouring the gel. The cell seeding well was punched such as to expose a section of the gradient (Fig. 1b). For the classic under-agarose assays, chemoattractant or vehicle solution was added in wells punched 2 mm away from the central well. We used fNLFNTK/FITC diluted in RPMI 1640 (10% FBS) at 200 nM. This chemotaxin diffuses through the gel generating a concentration gradient (Fig. 1c).

### Data acquisition

Typically, LDS751 dye-labeled neutrophils were imaged in epifluorescence every 5 seconds for approximately 2 hours with an excitation power of 300 µw (~1 nW per cell) during 100 ms, using a mCherry filter (excitation: 542–582 nm, emission: 603–683 nm). A custom-made LabVIEW program controlled a Retiga 2000R CCD camera attached to an inverted microscope (Olympus IX71, Japan) equipped with a 10x objective and a motorized stage. We obtained high throughput by multi-dimensional acquisition, programming the stage movement for sequential images at different locations of the sample. Throughout the assays, neutrophils were kept at 37 °C with 5% CO_2_ using a stage top incubator (Tokai Hit, Japan). Fluorescent formyl peptide patterns were imaged with a FITC filter (excitation: 463–505 nm, emission: 515–565 nm) using 2 s exposition time.

### Automated detection and tracking of neutrophils

We used MATLAB scripts (The MathWorks, MA, USA) for image analysis, neutrophil detection and tracking, as well as for describing cell trajectories and performing statistical analysis. The scripts are freely available upon request.

For each frame, we estimated the background intensity applying a morphological opening on the original image using a circular structuring element with twice the average diameter of neutrophils. We subtracted the background from the original image and enhanced the objects of neutrophil size by applying a band-pass filter. We defined the position of a cell at the highest peak within each region above a threshold^70^. For cell tracking, we employed an algorithm developed in-house^37^.

### Detection sensitivity

To assess the detection sensitivity, we acquired a set of data of 300 frames over 20 minutes, where cells where alternatively imaged either in bright field or fluorescence. The ground truth cell count for each frame was determined manually, using the bright field images data set. We calculated the sensitivity by dividing the true detected cell count by the ground true count.

### Gyration tensor analysis

We computed the gyration tensor to analyze cell migration dynamics. We analyzed the evolution of the gyration radius (R_g_), which is derived from the gyration tensor, as a function of time^71^. The gyration tensor is defined as

1 $$T=(\begin{array}{cc}{T}_{xx} & {T}_{xy}\\ {T}_{xy} & {T}_{yy}\end{array})=(\begin{array}{cc}{\langle x\rangle }^{2}-{\langle x\rangle }^{2} & \langle xy\rangle -\langle x\rangle \langle y\rangle \\ \langle xy\rangle -\langle x\rangle \langle y\rangle  & \langle {y}^{2}\rangle -{\langle y\rangle }^{2}\end{array})$$

where *x* and *y* are the coordinates of cell positions, and the operator <> indicates time average. R_g_ measures the spatial spread of cell positions within a trajectory and is computed as

2 $${R}_{g}^{2}={R}_{1}^{2}+{R}_{2}^{2}$$

where

3 $${R}_{1}^{2},{R}_{2}^{2}=\frac{1}{2}[({T}_{xx}+{T}_{yy})\pm \sqrt{{({T}_{xx}-{T}_{yy})}^{2}+4{T}_{xy}^{2}}]$$

are the Eigenvalues of *T*.

Small R_g_ values indicate a static period, since the cell positions are localized randomly around a same point. In contrast, during migration, the cell moves away from the starting position, yielding significantly larger R_g_ values. We have defined a time-normalised squared radius of gyration R_g_^2^_N_(t), which is computed using Eqs 1 and 2 by calculating the time average only over time frames later than t as:

4 $${\langle a\rangle }_{t}=\frac{{\sum }_{i=t}^{N}{a}_{i}}{N-t}$$

and dividing it by the number of frames involved in the calculation. The moment where R_g_^2^_N_ goes below a threshold determines when a cell reaches a static state.

Additionally, the parameter *A*_*2*_, derived from *T*, describes the elongation of the trajectory:

5 $${A}_{2}=\frac{{({R}_{1}^{2}-{R}_{2}^{2})}^{2}}{{({R}_{1}^{2}+{R}_{2}^{2})}^{2}}$$

Thus, an *A*_*2*_ close to 1 represents elongated trajectories, whereas A_2_ values around 0 indicate random walk trajectories. The time averages were computed as in Eq. 4.

We filtered the static end-segment of the tracks before calculating other parameters described afterwards. The thresholds for discarding end-segments were R_g_^2^_N_ < 0.1 μm^2^/frame and A_2_ < 0.6.

### Chemotactic indices

Forward Migration Index (FMI), the Side Migration Index (SMI), the total length (l_i_), the directness and the Chemotactic Precision Index were calculated as follows, using the terminology defined by Rink *et al*.^32^:

6 $$FMI=\frac{{y}_{end}-{y}_{1}}{{l}_{i}}$$

7 $$SMI=\frac{{x}_{end}-{x}_{1}}{{l}_{i}}$$

8 $$directness=\frac{{({({x}_{end}-{x}_{1})}^{2}+{({y}_{end}-{y}_{1})}^{2})}^{1/2}}{{l}_{i}}$$

where

9 $${l}_{i}={\sum }_{i=2}^{{n}_{steps}}{({({x}_{i}-{x}_{i-1})}^{2}+{({y}_{i}-{y}_{i-1})}^{2})}^{1/2}$$

10 $$CPI=\frac{FM{I}^{2}}{directness}\times (1-|SMI|)=directness\times co{s}^{2}\varphi \,\times (1-|SMI|)$$

where ϕ is the angle between FMI and directness. To standardise the comparison between the classic chemotaxis assay in diffusion and the haptotaxis assay, we chose to define the forward direction as the radial axis of the well, regardless of the direction of the substrate-bound gradient.

Mean cell speed within a trajectory and instant cell speed at any step point of a trajectory were calculated by dividing the total trajectory by the total time travelled (trajectory speed) and by dividing the length of each step by the time lag between frames (step speed). In order to evaluate the impact of the cell orientation on the speed, we also measure the angle between the direction of the immobilized gradient and the direction of each trajectory step.

### Penetration rate

To assess the rate at which cells enter the agarose-glass interstice, we counted, at consecutive intervals, tracks initiated nearby the well edge (within 100 µm from the edge), lasting at least 25 seconds, and showing elongation (R_g_^2^ > 40 μm^2^). We normalized the rate by the area occupied by the pattern.

### Statistical analysis

Data are presented as mean ± SD. Statistical comparisons were made by the Student’s test or the Mann-Whitney U test. For correlations, we calculated Pearson correlation coefficients (r). p < 0.05 was considered to be statistically significant for all tests. For histograms, error bars are calculated based on the propagation of uncertainty using the square root of the number of events, assuming that each bin regions represent a Poisson distribution of number of events^72^.

## Electronic supplementary material

Supplementary Info

Video 1 Classic under-agarose assay

Video 2 Haptotaxis

Video 3 Neutrophil morphology

Video 4 Neutrophil clustering

Video 5 Neutrophil penetration rate
