# Supplementary material for: A Haptotaxis Assay for Neutrophils using Optical Patterning and a High-content Approach
Source: Sci Rep. 2017 Jun 6;7:2869. doi: 10.1038/s41598-017-02993-6 (PMC5460230; doi:10.1038/s41598-017-02993-6)
Supplement: Supplementary file 1 — Supplementary Info [file 41598_2017_2993_MOESM1_ESM.pdf]

A Haptotaxis Assay for Neutrophils using Optical Patterning and High-content Approach

Joannie Roy, Javier Mazzaferri, János G. Filep, and Santiago Costantino

Supplementary Information

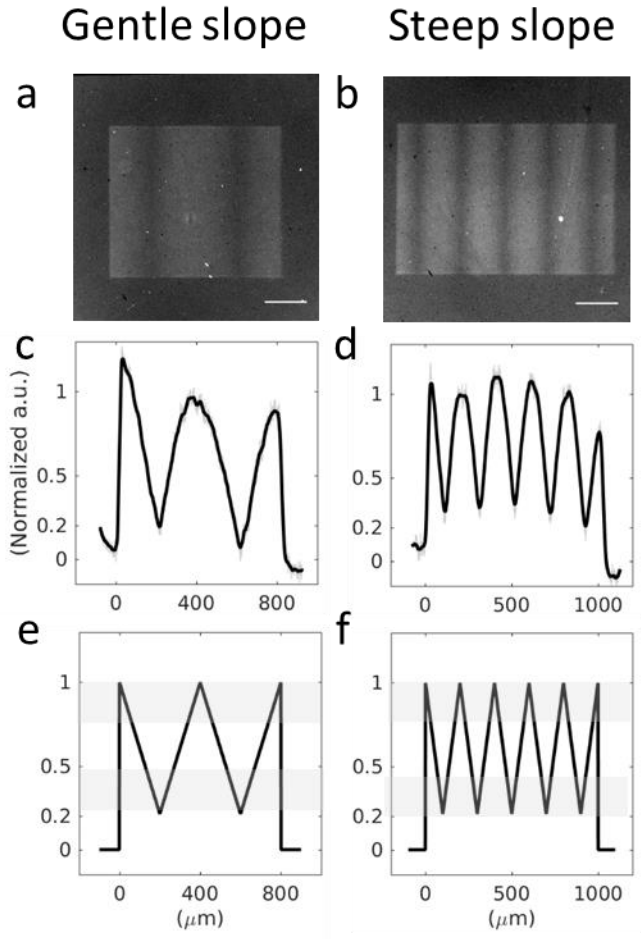

FIGURE S1. Characterization of surface-bound formyl-peptide gradients. (**a, c, and e**) Gentle gradient. (**b, d and f**) Steep gradient. (**a and b**) Fluorescent images of typical surface-bound gradients. Scale bar : 200 µm (**c and d**) Normalized fluorescent intensity profiles. In gray, raw data. In black, average-filtered smooth data. (**e and f**) Modeled gradient geometries. Steep gradient represent a difference of concentration between the front and the end of a 10 µm long neutrophil of 8% while the gentle, of 4%. The gray zones represent the 30% most and least concentrated part of the gradient.

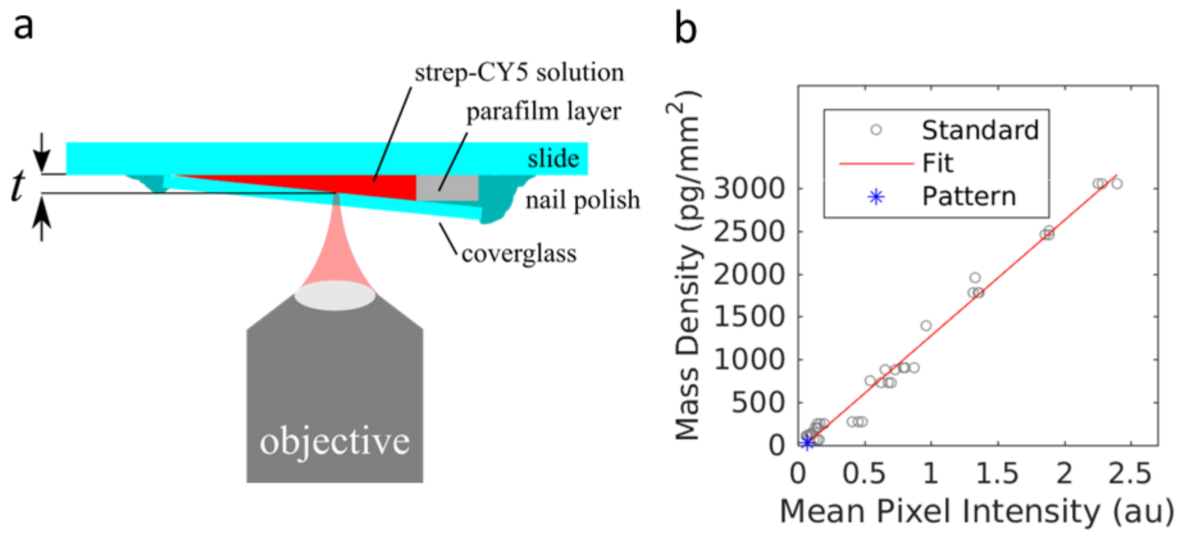

FIGURE S2. Quantification of bound molecules. (**a**) Wedge-shape glass chamber used to calibrate fluorescence intensity of standard solutions of Streptavidin-CY5. (**b**) Calibration data (Standard) used to perform a linear fit and calculate the mass density of a saturated pattern.

SUPPLEMENTARY METHOD:

We measured the number of molecules bound to the substrate in a LAPAP pattern by calibrating the fluorescence emission of Streptavidin-CY5 molecules. We fabricated a wedge-shaped glass chamber using a slide and a coverglass glued with nail polish, as depicted in Fig. S2 a. We filled the chamber with solutions of several concentrations of streptavidin-CY5, and imaged it at several regions using always the same camera settings. We measured the width of the chamber at each region using the calibrated screw of the microscope. Using the width of the chamber and the solution concentration, we computed the number of streptavidin-CY5 molecules per sample area, and we plotted this against the fluorescence emission per area, measured with the camera. Using a linear regression of these data and the fluorescence emission of the LAPAP pattern, we determined the average number of Streptavidin-CY5 molecules immobilized to the substrate, per unit area. Knowing the molecular weight of Steptavidin-CY5 to be 60kDa, we converted the calculated mass density of 23.67 pg/mm^2 to 23 770 molecules per 100µm^2, considering a typical neutrophil to be 10x10µm^2.

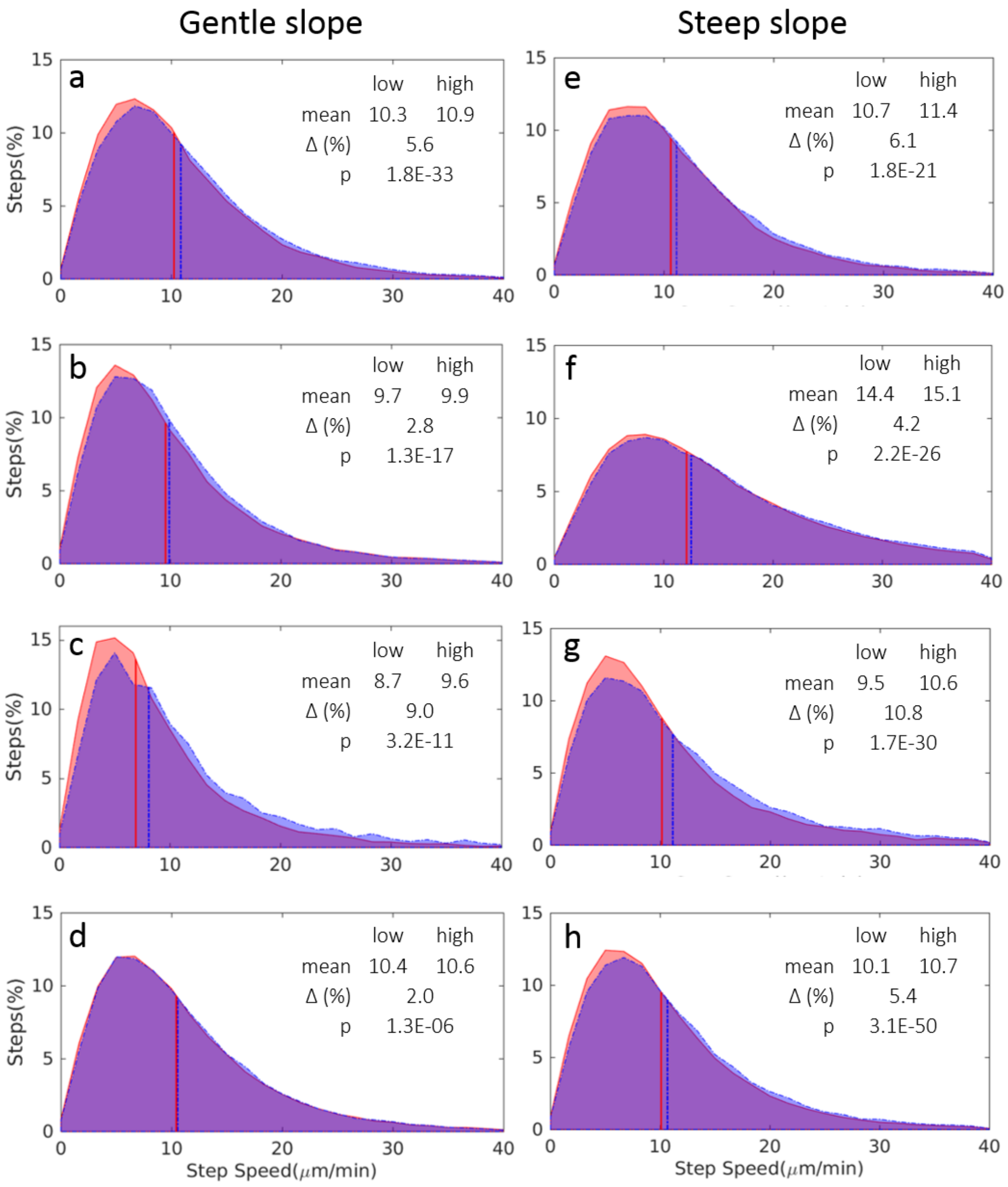

FIGURE S3. Impact of formyl peptide concentration on speed as a function of gradient slope. (**a-d**) Gentle slope (4% difference between the front and the back end of a cell). (**e-h**) Steep slope (8% difference between the front and the back end of a cell). Distribution of cell step speed over the 30% most concentrated zone of a gradient (high, blue) compared to those over the 30% least concentrated zone of the gradient (low, pink). Mean speed in μm/min. Percent difference of mean speed (Δ%). Individual data sets obtained for 8 independent experiments are shown. P-values were calculated using the Wilcoxon-Mann-Whitney test.

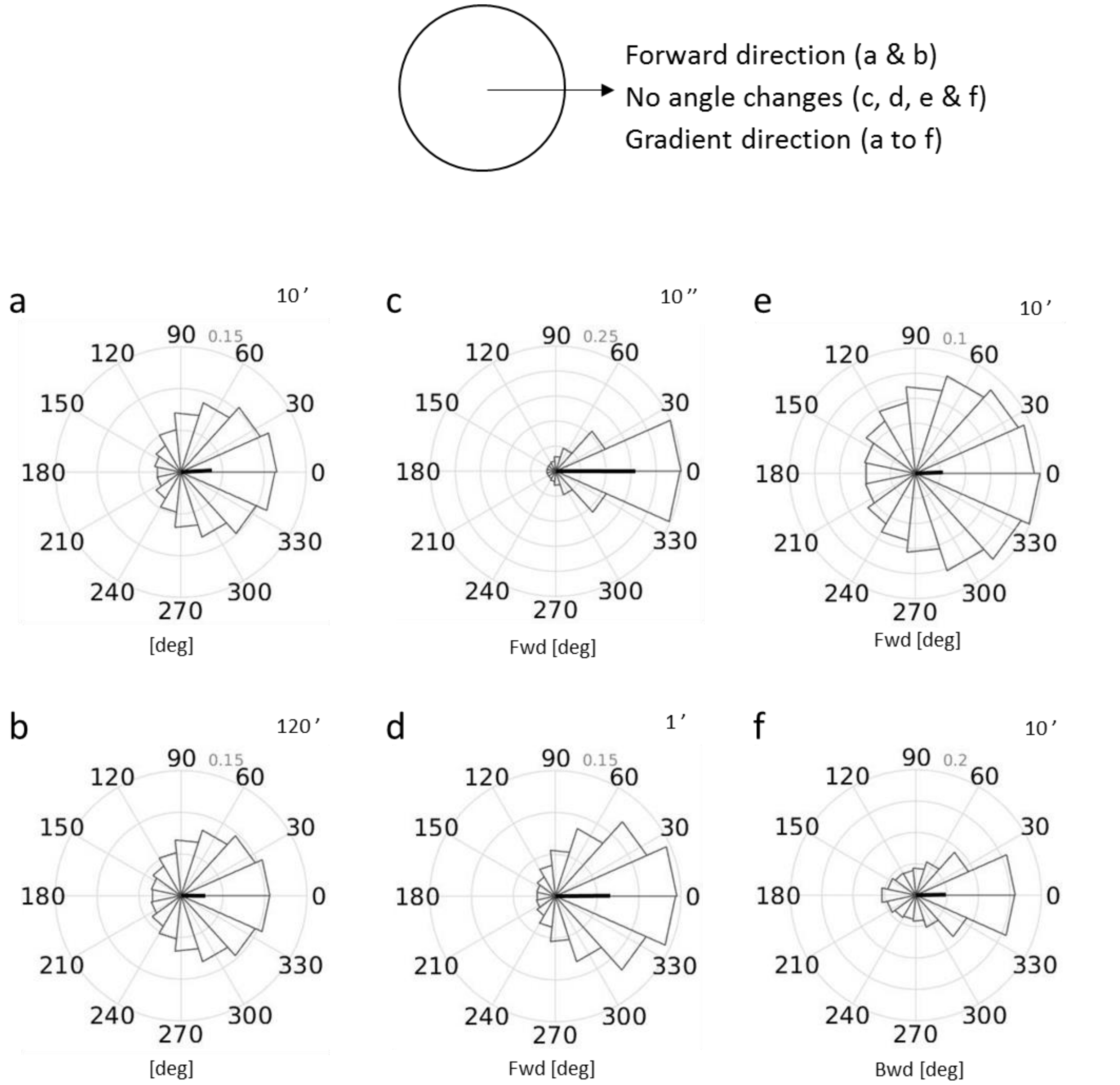

FIGURE S4. Cell orientation dynamics in the presence of soluble formyl peptide. (**a and b**) Distribution of step angles over 10 minutes (**a**) or 120 minutes (**b**). (**c, d, e and f**) Distribution of step angles differences after an initial forward step (**c, d, e**), or backward step (**f**) for delay times of 10 seconds, 1 minute and 10 minutes. The figure shows 1 experiment out of 2. Note that the persistence observed in f was inconsistent in the other experimental replicate.

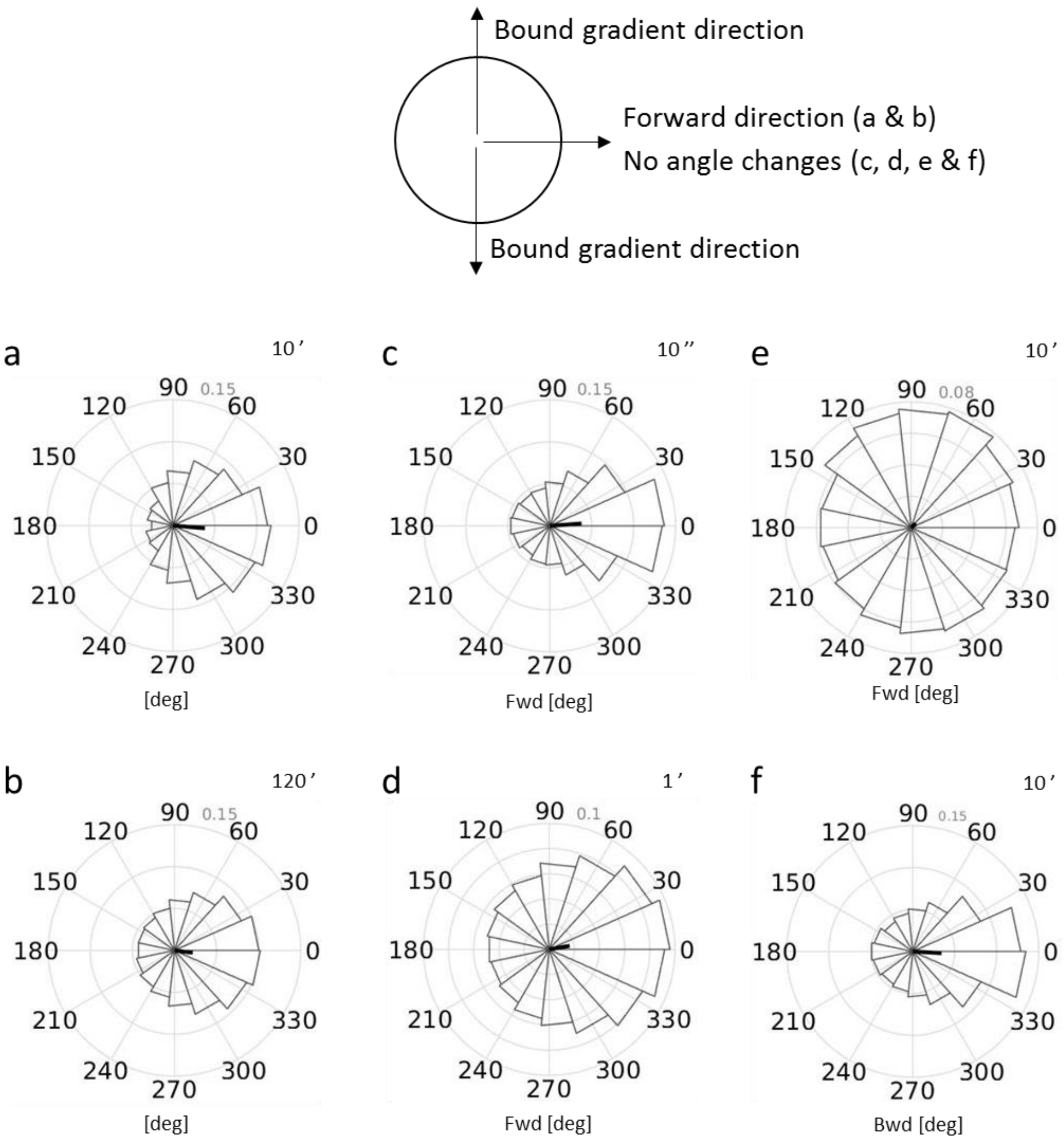

FIGURE S5. Cell orientation dynamics in the presence of bound formyl peptide. (**a and b**) Distribution of step angles over 10 minutes (**a**) or 120 minutes (**b**). (**c, d, e and f**) Distribution of step angles differences after an initial forward step (**c, d, e**), or backward step (**f**) for delay times of 10 seconds, 1 minute and 10 minutes. The figure shows 1 representative experiment out of 8.

Table I. Steps and tracks statistics for 12 individual experiments

**Replicate**	**# Steps**	**# Tracks**	**Track Length (**μm)	**Step Speed** (μm/min)
**Haptotaxis - Steep Slope (8%)**				
1	207079	2895	93 (± 89)	10.4 (± 6.9)
2	67867	934	84 (± 71)	10.8 (± 7.8)
3	259915	5448	99 (± 65)	14.1 (± 8.7)
4	95461	1187	107 (± 123)	10.9 (± 7.0)
**Haptotaxis - Gentle Slope (4%)**				
1	169712	2318	99 (± 95)	10.5 (± 7.0)
2	21263	182	98 (± 97)	9.2 (± 6.8)
3	138657	739	165 (± 171)	9.9 (± 6.7)
4	141773	2105	91 (± 89)	10.5 (± 6.7)
**Chemotaxis – Diffusion gradient**				
1 (fNLFNTK)	440762	5823	115 (± 100)	16.6 (± 9.6)
1 (vehicle)	48336	210	190 (± 209)	10.9 (± 7.5)
2 (fNLFNTK)	165878	3282	80 (± 59)	15.0 (± 8.8)
2 (vehicle)	26211	467	78 (± 68)	14.3 (± 8.4)

Values are the mean ± SD

Video Legends

Video S1. Classic under-agarose assay. Neutrophils were challenged with formyl peptides in solution. Left panel: field of view of neutrophils moving towards the source of fMLF. Right panel: neutrophil migration towards vehicle. Scale bar: 100 µm. The movie has been cropped and represents around 1/4 of the original field-of-view. Time label, hh:mm. The frame rate is 30 frames/second.

Video S2 Haptotaxis**.** Neutrophil migration on surface-bound formyl peptides gradients. Lines indicate regions of maximum to minimum concentration of immobilized formyl peptide. Arrows indicate the direction of the gradient. Scale bar: 100 µm. The movie has been cropped and represents around 1/2 of the original field-of-view. Time label, hh:mm. The frame rate is 30 frames/second.

Video S3. Neutrophil morphology**.** Bright field microscopy showing amoeboid-like morphology of neutrophils migrating under agarose towards vehicle solution (left panel), soluble fMLF (middle panel) or surface-bound formyl peptide gradient (right panel). Scale bar: 20 µm. Time label, hh:mm. The frame rate is 30 frames/second.

Video S4. Neutrophil clustering. Cells clustering on a 100 µm wide gradient. Lines indicate the limits of the gradient, and the circle indicates the center of a cluster. Time label, hh:mm The frame rate is 30 frames/second.

Video S5 Neutrophil Penetration Rate. Typical recording of neutrophils migrating on surface-bound formyl peptide (homogenous patterns). Top half: cells migrating on a 1/5 of peptide saturation. Bottom half: cells migrating on saturated peptides. Scale bar: 100 µm. Time label, mm:ss. The frame rate is 30 frames/second.
